# Interventions to Maintain HIV/AIDS, Tuberculosis, and Malaria Service Delivery During Public Health Emergencies in Low- and Middle-Income Countries: Protocol for a Systematic Review

**DOI:** 10.2196/64316

**Published:** 2025-01-15

**Authors:** Steven Ndugwa Kabwama, Rhoda K. Wanyenze, Helena Lindgren, Neda Razaz, John M Ssenkusu, Tobias Alfvén

**Affiliations:** 1 Department of Global Public Health Karolinska Institutet Stockholm Sweden; 2 Department of Community Health and Behavioral Sciences School of Public Health Makerere University Kampala Uganda; 3 Department of Disease Control and Environmental Health School of Public Health Makerere University Kampala Uganda; 4 Department of Women’s and Children’s Health Karolinska Institutet Stockholm Sweden; 5 Department of Medicine Karolinska Institutet Stockholm Sweden; 6 Department of Epidemiology and Biostatistics School of Public Health Makerere University Kampala Uganda; 7 Sachs’ Children and Youth Hospital Stockholm Sweden

**Keywords:** service availability, emergencies, tuberculosis, malaria, systematic reviews, health services, emergencies, HIV, AIDS, public health emergency, low- and middle-income countries, qualitative reviews, qualitative, policies, communities, health facilities, emergency, implement, implementation

## Abstract

**Background:**

Although existing disease preparedness and response frameworks provide guidance about strengthening emergency response capacity, little attention is paid to health service continuity during emergency responses. During the 2014 Ebola outbreak, there were 11,325 reported deaths due to the Ebola virus and yet disruption in access to care caused more than 10,000 additional deaths due to measles, HIV/AIDS, tuberculosis, and malaria. Low- and middle-income countries account for the largest disease burden due to HIV, tuberculosis, and malaria and yet previous responses to health emergencies showed that HIV, tuberculosis, and malaria service delivery can be significantly disrupted. To date, there has not been a systematic synthesis of interventions implemented to maintain the delivery of these services during emergencies.

**Objective:**

This study aimed to synthesize the interventions implemented to maintain HIV/AIDS, tuberculosis, and malaria services during public health emergencies in low- and middle-income countries.

**Methods:**

The systematic review was registered in the international register for prospective systematic reviews. It will include activities undertaken to improve human health either through preventing the occurrence of HIV, tuberculosis, or malaria, reducing the severity among patients, or promoting the restoration of functioning lost as a result of experiencing HIV, tuberculosis, or malaria during health emergencies. These will include policy-level (eg, development of guidelines), health facility–level (eg, service rescheduling), and community-level interventions (eg, community drug distribution). Service delivery will be in terms of improving access, availability, use, and coverage. We will report on any interventions to maintain services along the care cascade for HIV, tuberculosis, or malaria. Peer-reviewed study databases including MEDLINE, Web of Science, Embase, Cochrane, and Global Index Medicus will be searched. Reference lists from global reports on HIV/AIDS, tuberculosis, or malaria will also be searched. We will use the GRADE-CERQual (Grading of Recommendations Assessment, Development, and Evaluation—Confidence in Evidence from Reviews of Qualitative Research) approach to report on the quality of evidence in each paper. The information from the studies will be synthesized at the disease or condition level (HIV/AIDS, tuberculosis, and malaria), implementation level (policy, health facility, and community), and outcomes (improving access, availability, use, or coverage). We will use the PRISMA (Preferred Reporting Items for Systematic Reviews and Meta-Analyses) checklist to report findings and discuss implications for strengthening preparedness and response, as well as strengthening health systems in low- and middle-income countries.

**Results:**

The initial search for published literature was conducted between January 2023 and March 2023 and yielded 8119 studies. At the time of publication, synthesis and interpretation of results were being concluded. Final results will be published in 2025.

**Conclusions:**

The findings will inform the development of national and global guidance to minimize disruption of services for patients with HIV/AIDS, tuberculosis, and malaria during public health emergencies.

**Trial Registration:**

PROSPERO CRD42023408967; https://www.crd.york.ac.uk/prospero/display_record.php?RecordID=408967

**International Registered Report Identifier (IRRID):**

PRR1-10.2196/64316

## Introduction

### Background

The effective management of disasters requires deliberate investment across the phases of the disaster management cycle including mitigation or prevention, preparedness, response, and recovery [1]. In public health disaster management, several frameworks have been developed to provide a blueprint for investments to improve capacities for preparedness, response, and control of public health emergencies. The most widely applied framework is the World Health Organization (WHO) Joint External Evaluation of the International Health Regulations core capacities [2]. Others include the Global Health Security Index [3], the Health System Resilience Index [4], and the Epidemic Preparedness Index [5]. Taken together, the frameworks are comprehensive insofar as prescribing the mechanics or the hardware of a country’s capacity to respond to and control health emergencies. For example, investment in building diagnostic capacity, establishing emergency operations centers, investing in health workers, and having a coordination structure are all critical for both preparedness and response to emergencies. However, little attention is paid to issues such as leadership and trust [6], gender and equity [7], and continuity of other services [8]. Health service maintenance during public health emergencies is critical to minimize preventable disease mortality and morbidity. During the 2014 Ebola outbreak in West Africa, there were 11,325 reported deaths due to the Ebola virus [9] and yet the disruption in access to care also caused more than 10,000 additional preventable deaths due to measles, HIV/AIDS, tuberculosis, and malaria [10]. The 2009 H1N1 pandemic increased the risk of adverse neonatal outcomes among pregnant women [11] and increased emergency department visits [12]. The COVID-19 pandemic also reduced overall health care use across 20 countries by about a third [13]. Furthermore, a systematic review of the effects of the COVID-19 pandemic on service delivery and treatment outcomes of people living with HIV [14] noted that there were challenges in accessing services in health facilities, loss of adherence to drugs, and increase in mortality due to COVID-19–related complications. And yet, several modeling studies [15,16] predicted that even brief interruptions to services such as antiretroviral therapy (ART) and condoms could increase HIV/AIDS–related morbidity and mortality in both the short and long term. Previous researchers have described this failure as the “tyranny of the urgent” [7,17] where the response to the public health emergency is prioritized over other health system functions like routine service delivery. In fact, prior to the COVID-19 pandemic, service continuity was not part of the standard operations of an emergency response [18], and the WHO monitoring and evaluation framework for assessing the effectiveness of a response did not have indicators for monitoring continuity of delivery of other services [19]. Our aim is not to contend that services are delivered during the emergency in the exact same way as in normal settings but to recognize the need for modifications and adaptations to service delivery to maintain demand for and continued access to services. Since the WHO International Health Regulations (2005) governing framework for global health security was established [20], the events that have been declared public health emergencies of international concern include the H1N1 pandemic influenza of 2009, Ebola outbreak in West Africa (2013-2015), Ebola outbreak in the Democratic Republic of Congo (2018-2020), Zika virus outbreak (2016), poliomyelitis (2014 to present), the COVID-19 pandemic [21], and the monkeypox outbreak (2022-2023). Previous studies have consistently shown the impact these public health emergencies have on access to HIV, tuberculosis, and malaria services [22-26]; in addition to the already high mortality and morbidity due to these diseases in low- and middle-income countries [27]. To date, there has not, to our knowledge, been a systematic synthesis of the interventions implemented to maintain the demand for and delivery of HIV, tuberculosis, and malaria services during emergencies. Recent reviews have primarily focused on the challenges [28] or the interventions [29] for the delivery of maternal and child health services during emergencies. The findings from this review will inform the development of national and global guidance on the maintenance of services for HIV/AIDS, tuberculosis, and malaria during public health emergencies.

### Review Question

What interventions have been implemented to maintain the delivery of HIV/AIDS, tuberculosis, and malaria services during public health emergencies in low- and middle-income countries?

### Objective

This study aims to identify strategies and interventions used to maintain the delivery of and access to HIV/AIDS, tuberculosis, and malaria services during public health emergencies of international concern including H1N1 2009, Ebola 2014, Zika 2015, and the COVID-19 pandemic in low- and middle-income countries to inform efforts for incorporating essential health services maintenance as a key preparedness and response strategy.

## Methods

### Study Design

The proposed systematic review will be conducted following the guidance given by the Cochrane Handbook for Systematic Reviews of Interventions [30]. The systematic review has been registered in the International Register for Prospective Systematic Reviews (PROSPERO Registration #CRD42023408967).

### Inclusion Criteria

#### Concept

For purposes of this systematic review, we will define interventions as any strategies or activities undertaken to improve human health either through preventing the occurrence of HIV, tuberculosis, or malaria, reducing the severity of HIV, tuberculosis, or malaria among patients, or promoting the restoration of functioning lost as a result of experiencing HIV, tuberculosis, or malaria [31]. The interventions for the continuity of HIV/AIDS, tuberculosis, and malaria services will include policy-level interventions (eg, development of guidelines), health facility–level interventions (eg, service rescheduling), and community-level interventions (eg, community drug distribution). We will define service delivery in terms of improving access (physical and economic ability to use services), availability (physical presence of services), use (quantity of services used), and coverage (proportion of people who access a needed service) [32].

We will report on any interventions to maintain services along the stages of the HIV care cascade including improving testing, linkage to care, retention in care, ART adherence, and viral suppression [33]. Similarly, we will report any interventions implemented to maintain services along any of the stages of the tuberculosis care cascade including tuberculosis testing, linkage to treatment, retention in treatment, posttreatment survival, and achieving durable cure [34]. For malaria, we will include interventions aimed at prevention (eg, vector control), diagnosis, or treatment according to the WHO malaria guidelines [35]. We will include studies reporting any quantitative assessment of service delivery processes (screening, testing, linkage to care, retention in care, and ART adherence) or outcomes (viral suppression, cure, and case counts).

#### Context

This systematic review will include studies conducted to maintain delivery of and access to HIV, tuberculosis, and malaria services during public health emergencies of international concern in low- and middle-income countries according to the World Bank classification of countries by income level [36].

### Types of Sources

This systematic review will consider both quantitative and qualitative studies that use cross-sectional, case-control, randomized and nonrandomized control trials, or cohort study designs. Other designs such as before and after, interrupted time series, and analytical observational studies will be considered for inclusion. Global disease-specific reports published by the Joint United Nations Programme on HIV/AIDS, WHO, or any other global bodies will be considered for inclusion. Editorials, preprints, abstracts, study protocols, commentaries, webinar papers, or opinion pieces will not be eligible for inclusion in the study. Studies conducted among animals or studies assessing the direct effects of emergencies will also be excluded as the review focuses on the maintenance of services for HIV/AIDS, tuberculosis, and malaria.

### Search Strategy

A test search was conducted in PubMed to identify published papers on the maintenance of HIV, tuberculosis, and malaria services during public health emergencies. Thereafter, we developed a full search using words in the titles and abstracts of the papers we identified to be relevant (Multimedia Appendix 1). The search strategy will be used to identify relevant papers in each of the other databases. We will include only studies published in English. In addition, we will restrict the search to studies published between 2009 (when the H1N1 pandemic happened) and the end of March 2023.

Peer-reviewed study databases including MEDLINE, Web of Science, Embase, Cochrane, and Global Index Medicus will be searched. Two experienced librarians at Karolinska Institutet’s University Library provided guidance about the choice of article databases that would provide the most relevant studies related to the research question. In addition, we will review reference lists of eligible studies, specific journals, and Google Scholar for relevant studies. Reference lists from Global Reports for HIV/AIDS, tuberculosis, or malaria will also be searched for relevant papers. The search will be guided by 2 experienced librarians from Karolinska Institutet’s University Library. We anticipate that this search will be completed by the end of May 2023.

After the search is completed, the citations of all identified studies will be exported to Endnote X7 (Clarivate) and screened to remove any duplicates. Thereafter, the search will then be exported to Rayyan [37]—a web-based software that was developed by the Qatar Computing Research Institute and uses semiautomation to expedite the process of screening studies. Two reviewers will independently apply the inclusion and exclusion criteria and select studies based on the title and abstract. Disagreements between the individual judgments of the reviewers will be resolved through a discussion. After consensus building on studies based on titles and abstracts, additional studies may be excluded at full-text review.

### Data Extraction

A data abstraction form will be used to obtain information from each of the relevant studies (Multimedia Appendix 2). The data to be extracted from each paper includes the year of publication, first author, year of study, journal or other publication source, country or countries, title, study design, target population, circumstance or outbreak, disease category (HIV/AIDS, tuberculosis, malaria, or a combination of these), list of measures implemented to maintain services, and limitations and strengths. The other information to be extracted about the interventions include the implementer (facility health workers and community health workers), the level at which the intervention is implemented (facility and community), the measure of assessment of the intervention (eg, number of people initiated on ART, number of people tested, and viral load coverage), and the type of indicator (process: number receiving drugs or outcome: number virally suppressed). The number of variables and the order in which they are presented will be adjusted depending on the amount of relevant information we find in the identified studies.

#### Risk of Bias or Quality Assessment

We will use several strategies to ensure the quality of studies included in the systematic review. First, we will involve 2 reviewers to screen the studies that will be included in the review. The reviewers will have experience in research related to HIV/AIDS, tuberculosis, and malaria service delivery as well as response and control of public health emergencies. In addition, since we do not anticipate quantitatively combining the relevant studies, we will use the GRADE-CERQual (Grading of Recommendations Assessment, Development, and Evaluation—Confidence in Evidence from Reviews of Qualitative Research) approach [38] to assess the quality of each paper. Each study will be assessed for quality in terms of the methodological quality, coherence, adequacy, and quality of the data collected and the relevance of results in line with the review question. The overall assessment of the quality of each study will be graded as either high, moderate, low, or very low.

#### Data Synthesis and Presentation

Findings from the systematic review will be presented using the PRISMA (Preferred Reporting Items for Systematic Reviews and Meta-analyses) checklist and flow diagram [39]. The data extracted for the review studies will be summarized and presented in tabular or graphic form as appropriate. The synthesis of the data will be conducted at various levels. These will include at disease or condition level (HIV/AIDS, tuberculosis, malaria, or a combination of these), at the implementation level (policy, health facility, and community), and outcomes (improving access, availability, use, or coverage of services)

## Results

The initial search for published literature yielded 8119 studies from web-based databases. This search was conducted between January 2023 and March 2023. At the time of publication, synthesis and interpretation of results were being concluded. Final results will be published in 2025.

## Discussion

### Expected Findings

We will discuss the findings and their implications for strengthening both preparedness and response, as well as strengthening health systems in low- and middle-income countries. Specifically, we will discuss interventions that are common to the 3 disease programs and how such interventions can support the response to a public health emergency but also be scaled up to strengthen service delivery beyond the emergency. We will also discuss how the interventions have been assessed by comparing process indicators (such as number of people tested) and outcome indicators (such as number of people with viral suppression).

Previous literature has tried to synthesize the challenges [28] and interventions [29] for the delivery of specific service delivery programs like delivery of maternal and child health services during emergencies. However, the scope of essential health services is broad and includes services for noncommunicable diseases like diabetes and hypertension, services for older adults as well as health promotion and referral services. This protocol will provide a structure and framework for the synthesis of the literature for the continuity of essential health services more broadly beyond HIV/AIDS, tuberculosis, and malaria.

### Limitations

The main limitation of the review will be the exclusion of studies conducted in languages other than English. In addition, the review will include only studies publishing information about interventions implemented during public health emergencies of international concern. However, a public health emergency need not be declared to be of international concern to cause disruption of access and delivery of HIV, tuberculosis, and malaria services. Nonetheless, the identified interventions will be transferrable to contexts in which an emergency is local and does not pose a threat to the international community. In addition, the development of the protocol followed the PRISMA-P (Preferred Reporting Items for Systematic Reviews and Meta-Analysis Protocols) checklist (Multimedia Appendix 3).

### Conclusions

The findings will inform the development of national and global guidance to minimize disruption of services for patients with HIV/AIDS, tuberculosis, and malaria during public health emergencies. The protocol will also inform studies conducted to synthesize interventions to maintain other health programs beyond HIV/AIDS, tuberculosis, and malaria.

## Appendix

Multimedia Appendix 1 Search strategy.

Multimedia Appendix 2 Data Abstraction Form.

Multimedia Appendix 3 Preferred Reporting Items for Systematic Reviews and Meta-Analysis Protocols (PRISMA-P) checklist.

## Abbreviations

ART: antiretroviral therapy
CERQual: Confidence in Evidence from Reviews of Qualitative Research
GRADE: Grading of Recommendations Assessment, Development, and Evaluation
PRISMA: Preferred Reporting Items for Systematic Reviews and Meta-analyses
PRISMA-P: Preferred Reporting Items for Systematic Reviews and Meta-Analysis Protocols
PROSPERO: International Register for Prospective Systematic Reviews
WHO: World Health Organization
