# Supplementary material for: Interventions to Maintain HIV/AIDS, Tuberculosis, and Malaria Service Delivery During Public Health Emergencies in Low- and Middle-Income Countries: Protocol for a Systematic Review
Source: JMIR Res Protoc. 2025 Jan 15;14:e64316. doi: 10.2196/64316 (PMC11780283; doi:10.2196/64316)
Supplement: Multimedia Appendix 1 [file resprot_v14i1e64316_app1.pdf]

Documentation of search strategies
University Library search consultation group

Date: March 2023

Topic/research question: Essential health service delivery during public health emergencies

Name of researcher(s): Steven Ndugwa Kabwama, Global public health

Librarian(s): Emma-Lotta Säätelä & Love Strandberg

Databases:

1. Medline (Ovid)
2. Embase (embase.com)
3. Web of Science Core Collection, CABI and Scielo via Web of Science (Clarivate)
4. Cochrane Library (Wiley)
5. Global Index Medicus (globalindexmedicus.net)

Total number of hits:

- Before deduplication: 8,119
- After deduplication: 5,943

Text that can be used in the Methods-section:

<u>Search strategy</u>

A literature search was performed in the following databases: Medline (Ovid), Embase (embase.com), Cochrane Library (Wiley), Global Index Medicus and Web of Science Core Colletcion, Scielo and CABI CAB Asbtracts & Global Health via Web of Science (clarivate). The last search was conducted 2023-03-31.

The search strategy was developed in Medline (Ovid) in collaboration with librarians at the Karolinska Institutet University Library. For each search concept Medical Subject Headings (MeSH-terms) and free text terms were identified. The search was then translated, in part using Polyglot Search Translator (1), into the other databases.

No language restriction was applied and databases were searched from inception. Preprints, conference abstracts and clinical trial protocols were excluded from the search.

The strategies were peer reviewed by another librarian prior to execution.

De-duplication was done using the method described by Bramer et al (2). One final, extra step was added to compare DOIs.

A snow-ball search was applied to check references and citations of eligible studies from the database searches using XX [specify which resource was used for checking references/citations].

The full search strategies for all databases are available in the appendix.

References

1. Clark JM, Sanders S, Carter M, Honeyman D, Cleo G, Auld Y, Booth D, Condron P, Dalais C, Bateup S, Linthwaite B, May N, Munn J, Ramsay L, Rickett K, Rutter C, Smith A, Sondergeld P, Wallin M, Jones M, Beller E. (2020) Improving the translation of search strategies using the Polyglot Search Translator: a randomized controlled trial. *Journal of the Medical Library Association: JMLA*. 108(2):195-207. doi: 10.5195/jmla.2020.834.

2. Bramer, W. M., Giustini, D., de Jonge, G. B., Holland, L., & Bekhuis, T. (2016). De-duplication of database search results for systematic reviews in EndNote. *Journal of the Medical Library Association: JMLA*, 104(3), 240-243. doi: 10.3163/1536-5050.104.3.014

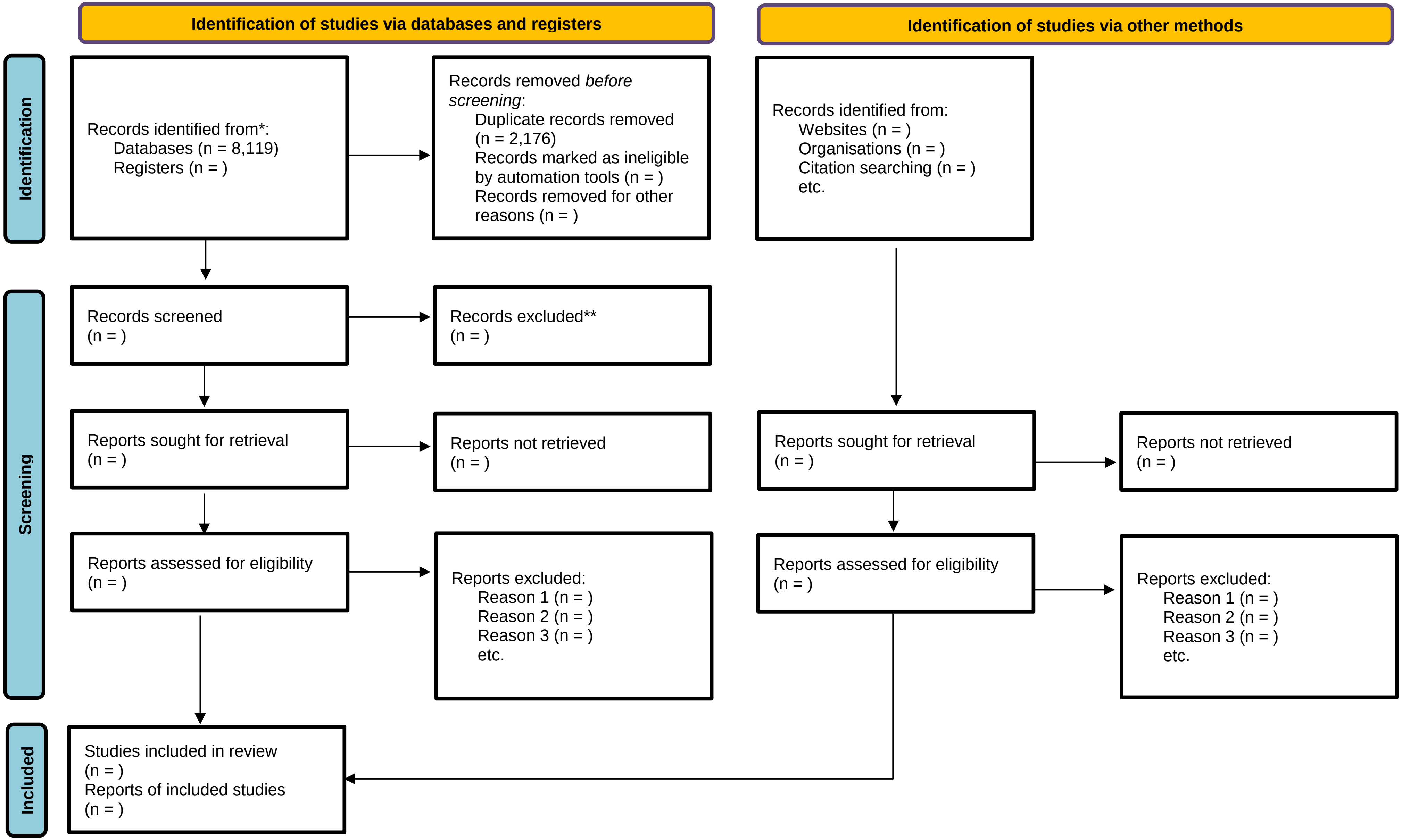

*Consider, if feasible to do so, reporting the number of records identified from each database or register searched (rather than the total number across all databases/registers).
**If automation tools were used, indicate how many records were excluded by a human and how many were excluded by automation tools.

From: Page MJ, McKenzie JE, Bossuyt PM, Boutron I, Hoffmann TC, Mulrow CD, et al. The PRISMA 2020 statement: an updated guideline for reporting systematic reviews. BMJ 2021;372:n71. doi: 10.1136/bmj.n71. For more information, visit: http://www.prisma-statement.org/

1. Medline

Interface: Ovid MEDLINE(R) and Epub Ahead of Print, In-Process & Other Non-Indexed Citations and Daily<br><br>Date of Search: 31 March 2023<br><br>Number of hits: 2,970<br><br>Comment: In Ovid, two or more words are automatically searched as phrases; i.e. no quotation marks are needed	Field labels<br><br>• exp/ = exploded MeSH term<br>• / = non exploded MeSH term<br>• .ti,ab,kf. = title, abstract and author keywords<br>• adjx = within x words, regardless of order<br>• * = truncation of word for alternate endings<br>• ?=0-1 character(s)

Database(s): **Ovid MEDLINE(R) ALL** 1946 to March 30, 2023
Search Strategy:

#	Searches	Results
1	Medically Underserved Area/ or Developing Countries/ or Rural Health/ or Rural Population/	172492
2	Afghanistan/ or Albania/ or Algeria/ or Angola/ or Argentina/ or Armenia/ or Azerbaijan/ or Bangladesh/ or Benin/ or "Republic of Belarus"/ or Belize/ or Bhutan/ or Bolivia/ or "Bosnia and Herzegovina"/ or Botswana/ or Brazil/ or Bulgaria/ or Burkina Faso/ or Burundi/ or Cambodia/ or Cameroon/ or Cabo Verde/ or Central African Republic/ or Chad/ or exp China/ or Colombia/ or Comoros/ or Congo/ or Costa Rica/ or Cote d'Ivoire/ or Cuba/ or Djibouti/ or Dominican Republic/ or Ecuador/ or Egypt/ or El Salvador/ or Eritrea/ or Equatorial Guinea/ or Ethiopia/ or Fiji/ or Gabon/ or Gambia/ or Georgia/ or Ghana/ or Grenada/ or Guatemala/ or Guinea/ or Guinea-Bissau/ or Guyana/ or Haiti/ or Honduras/ or India/ or Indonesia/ or Iran/ or Iraq/ or Jamaica/ or Jordan/ or Kazakhstan/ or Kenya/ or "Democratic People's Republic of Korea"/ or Kosovo/ or Kyrgyzstan/ or Laos/ or Lebanon/ or Lesotho/ or Liberia/ or Libya/ or Macedonia/ or Madagascar/ or Malaysia/ or Malawi/ or Mali/ or Mauritania/ or Micronesia/ or Mauritius/ or Mexico/ or Moldova/ or Mongolia/ or Montenegro/ or Morocco/ or Mozambique/ or Myanmar/ or Namibia/ or Nepal/ or Nicaragua/ or Niger/ or Nigeria/ or Pakistan/ or Palau/ or "Papua New Guinea"/ or Paraguay/ or Peru/ or Philippines/ or Romania/ or Russia/ or Rwanda/ or Saint Lucia/ or "Saint Vincent and the Grenadines"/ or "Independent State of Samoa"/ or "Sao Tome and Principe"/ or Senegal/ or Serbia/ or Sierra Leone/ or exp Melanesia/ or Sri Lanka/ or Somalia/ or South Sudan/ or Sudan/ or South Africa/ or Suriname/ or Swaziland/ or Syria/ or Tajikistan/ or Tanzania/ or Timor-Leste/ or Thailand/ or Togo/ or Tonga/ or Tunisia/ or Turkey/ or Turkmenistan/ or Uganda/ or Ukraine/ or exp USSR/ or Uzbekistan/ or Vanuatu/ or Venezuela/ or Vietnam/ or Yemen/ or Zambia/ or Zimbabwe/	1262300

3	(Afghanistan or Albania* or Algeria* or American Samoa or Angola* or Argentina or Armenia* or Azerbaijan or Bangladesh or Benin or Byelarus or Byelorussian or Belarus or Belorussian or Belorussia or Belize or Bhutan or Bolivia* or Bosnia* or Herzegovina or Hercegovina or Botswana or Brazil* or Brasil* or Bulgaria* or Burkina Faso or Burkina Fasso or Burundi or Urundi or Cambodia* or Khmer Republic or Kampuchea or Cameroon or Cameroons or Cameron or Camerons or Cape Verde or Cabo Verde or Central African Republic or Chad or China or Colombia* or Comoros or Comoro Islands or Comores or Congo or Costa Rica or Cote d'Ivoire or Ivory Coast or Cuba or Djibouti or Dominica or Dominican Republic or East Timor or East Timur or Timor Leste or Ecuador or Egypt* or El Salvador or Eritrea* or Equatorial Guinea or Eswatini* or Ethiopia* or Fiji or Gabon or Gambia* or Gaza or Georgia Republic or Georgian Republic or Ghana or Grenada or Guatemala* or Guinea or Guyana or Haiti or Honduras or India or Indonesia* or Iran or Iraq or Jamaica* or Jordan* or Kazakhstan or Kenya* or Kiribati or Democratic People's Republic of Korea* or North Korea* or Kosovo or Kyrgyz Republic or Lao PDR or Laos or Lebanon or Lesotho or Liberia* or Libya* or Macedonia* or Madagascar or Malaysia* or Malaya* or Malay or Malawi or Mali or Maldives or Marshall Islands or Mauritania* or Mauritius or Mexico or Mehico or Micronesia* or Middle East or Moldova or Moldovia* or Moldovian* or Mongolia or Montenegro or Morocco or Mozambique or Mocambique or Myanmar or Namibia* or Nauru or Nepal or Nicaragua or Niger or Nigeria* or Pakistan* or Palau or Palestine* or Paraguay or Peru* or Philippines or Philipines or Phillipines or Phillippines or Romania* or Rumania* or Roumania* or Russia or Russian or Rwanda or Ruanda or Saint Lucia or St Lucia or Saint Vincent or St Vincent or Grenadines or Samoa or Samoan Islands or Sao Tome or Senegal or Serbia* or Sierra Leone or Spanish Guinea or Sri Lanka or Ceylon or Solomon or USSR or Soviet Union or "Union of Soviet Socialist Republics" or Somalia* or South Africa* or Sudan or Suriname or Surinam or Swaziland or Syria or Syrian Arab Republic or Tajikistan or Tadzhikistan or Tadjikistan or Tadzhik or Tanzania or Thailand or Togo or Togolese Republic or Tonga or Tunisia* or Turkey or Turkiye or Turkmenistan or Tuvalu or Uganda or Ukrain* or Uzbekistan or Uzbek or Vanuatu or Venezuela or Vietnam or Viet Nam or West Bank or Yemen or Zambia or Zimbabwe).ti,ab,kf.	1519940
4	((developing or emerging or less* developed or under developed or underdeveloped or middle income or low* income or third-world or underserved or under served or deprived or poor*) adj3 (countr* or nation? or population? or world or economy or economies)).ti,ab.	150412
5	(low* adj2 (countr* or gdp or gnp or gross domestic or gross national)).ti,ab.	16048
6	((low* or middle*) adj5 (countr* or nation*)).ti,ab,kf.	65503

7	((rural or remote or nonmetropolitan or non-metropolitan or underserved or under served or deprived or shortage) adj (communit$ or count$ or area? or region? or province? or district?)).ti,ab.	83432
8	(Global South or LIC or LMIC* or LMICs or MIC or South-South or rural health* or rural population*).ti,ab,kf.	83831
9	or/1-8	2182102
10	Hemorrhagic Fever, Ebola/	6588
11	Ebolavirus/	3947
12	Ebola vaccines/	763
13	Zika virus infection/	6880
14	Zika virus/	6309
15	Influenza A Virus, H1N1 Subtype/	17387
16	(ebola or zika or zikv or h1n1*).ti,ab,kf.	41030
17	(swine and (flu or influenza)).ti,ab,kf.	4886
18	COVID-19/	216857
19	exp COVID-19 Testing/	10796
20	exp COVID-19 Vaccines/	20344
21	(nCoV* or 2019nCoV or 19nCoV or COVID19* or COVID or SARS-COV-2 or SARSCOV-2 or SARS-COV2 or SARSCOV2 or SARS coronavirus 2 or Severe Acute Respiratory Syndrome Coronavirus 2 or Severe Acute Respiratory Syndrome Corona Virus 2).ti,ab,kf.	335614
22	or/10-21	390968
23	exp HIV Infections/	313376
24	exp HIV/	107025
25	exp Tuberculosis/	205432
26	exp Malaria/	73892
27	exp Antitubercular Agents/	97894
28	Tuberculin Test/	14275
29	Antimalarials/	28896
30	exp HIV testing/	7562
31	exp Anti-HIV agents/	75457
32	(malaria* or antimalaria* or plasmodium or HIV or AIDS or tubercul* or antitubercul* or immun* deficienc* or immunodeficienc*).ti,ab,kf.	851639
33	or/23-32	971151

Karolinska Institutet

34	9 and 22 and 33	2970

Karolinska Institutet

2. Embase

Interface: embase.com<br><br>Date of Search: 31 March 2023<br><br>Number of hits: 3,523<br><br>Comment: Emtree is the controlled vocabulary in Embase	Field labels<br><br>• /exp = exploded Emtree term<br>• /de = non exploded Emtree term<br>• ti,ab,kw = title, abstract and author keywords<br>• NEAR/x = within x words, regardless of order<br>• * = truncation of word for alternate endings

No.	Query	Results
**#38**	**#36** NOT **#37**	**3,523**
**#37**	**#8** AND **#21** AND **#33** AND [preprint]/lim	**65**
**#36**	**#34** NOT **#35**	**3,588**
**#35**	**#8** AND **#21** AND **#33** AND ([conference abstract]/lim OR [conference paper]/lim OR [conference review]/lim)	**768**
**#34**	**#8** AND **#21** AND **#33**	**4,356**
**#33**	**#22** OR **#23** OR **#24** OR **#25** OR **#26** OR **#27** OR **#28** OR **#29** OR **#30** OR **#31** OR **#32**	**1,219,670**
**#32**	**malaria***:ti,ab,kw OR **antimalaria***:ti,ab,kw OR **plasmodium**:ti,ab,kw OR **hiv**:ti,ab,kw OR **aids**:ti,ab,kw OR **tubercul***:ti,ab,kw OR **antitubercul***:ti,ab,kw OR **'immun* deficienc*'**:ti,ab,kw OR **immunodeficienc***:ti,ab,kw	**1,046,087**
**#31**	**'antimalarial agent'**/de	**28,612**
**#30**	**'malaria'**/exp	**109,483**
**#29**	**'tuberculostatic agent'**/de	**43,931**

**#28**	**'tuberculin test'**/de	**23,758**
**#27**	**'tuberculosis'**/exp	**294,486**
**#26**	**'human immunodeficiency virus vaccine'**/de OR **'anti human immunodeficiency virus agent'**/de	**35,560**
**#25**	**'hiv test'**/exp	**15,505**
**#24**	**'acquired immune deficiency syndrome'**/exp	**153,813**
**#23**	**'human immunodeficiency virus infection'**/exp	**425,233**
**#22**	**'human immunodeficiency virus'**/exp	**217,380**
**#21**	**#9** OR **#10** OR **#11** OR **#12** OR **#13** OR **#14** OR **#15** OR **#16** OR **#17** OR **#18** OR **#19** OR **#20**	**455,192**
**#20**	**swine**:ti,ab,kw AND (**flu**:ti,ab,kw OR **influenza**:ti,ab,kw)	**5,689**
**#19**	**ebola**:ti,ab,kw OR **zika**:ti,ab,kw OR **zikv**:ti,ab,kw OR **h1n1***:ti,ab,kw	**50,016**
**#18**	**ncov***:ti,ab,kw OR **2019ncov**:ti,ab,kw OR **19ncov**:ti,ab,kw OR **covid19***:ti,ab,kw OR **covid**:ti,ab,kw OR **'sars cov 2'**:ti,ab,kw OR **'sarscov 2'**:ti,ab,kw OR **'sars cov2'**:ti,ab,kw OR **sarscov2**:ti,ab,kw OR **'sars coronavirus 2'**:ti,ab,kw OR **'severe acute respiratory syndrome coronavirus 2'**:ti,ab,kw OR **'severe acute respiratory syndrome corona virus 2'**:ti,ab,kw	**368,161**
**#17**	**'covid-19 testing'**/exp	**9,003**
**#16**	**'sars-cov-2 vaccine'**/exp	**33,667**
**#15**	**'coronavirus disease 2019'**/exp	**303,478**

**#14** 'influenza a (h1n1)'/de OR '2009 h1n1 influenza'/de	12,587
**#13** 'zika virus vaccine'/de	628
**#12** 'zika fever'/de	8,781
**#11** 'zika virus'/exp	9,000
**#10** 'ebola vaccine'/de	1,419
**#9** 'ebola hemorrhagic fever'/de	8,845
**#8** #1 OR #2 OR #3 OR #4 OR #5 OR #6 OR #7	2,083,782
**#7** 'global south':ti,ab,kw OR lic:ti,ab,kw OR lmic*:ti,ab,kw OR lmics:ti,ab,kw OR mic:ti,ab,kw OR 'south south':ti,ab,kw OR 'rural health*':ti,ab,kw OR 'rural population*':ti,ab,kw	107,754
**#6** ((rural OR remote OR nonmetropolitan OR 'non metropolitan' OR underserved OR 'under served' OR deprived OR shortage) NEXT/1 (communit? OR count? OR area$ OR region$ OR province$ OR district$)):ti,ab	89,872
**#5** ((low* OR middle*) NEAR/5 (countr* OR nation*)):ti,ab,kw	80,540
**#4** (low* NEAR/2 (countr* OR gdp OR gnp OR 'gross domestic' OR 'gross national')):ti,ab	20,220
**#3** africa:ti,ab OR asia:ti,ab OR caribbean:ti,ab OR 'west indies':ti,ab OR 'south america':ti,ab OR 'latin america':ti,ab OR 'central america':ti,ab	308,078
**#2** 'afghanistan'/de OR 'albania'/de OR 'algeria'/de OR 'angola'/de OR 'argentina'/de OR 'armenia'/de OR 'azerbaijan'/de OR 'bangladesh'/de OR 'benin'/de OR 'belarus'/de OR 'belize'/de OR 'bhutan'/de OR 'bolivia'/de OR 'bosnia and herzegovina'/exp OR 'botswana'/de OR 'brazil'/exp OR 'bulgaria'/de	1,651,978

OR **'burkina faso'**/de OR **'burundi'**/de OR **'cambodia'**/de OR **'cameroon'**/de OR **'cape verde'**/de OR **'central african republic'**/de OR **'chad'**/de OR **'china'**/exp OR **'colombia'**/de OR **'comoros'**/de OR **'congo'**/de OR **'costa rica'**/de OR **'cote divoire'** OR **'cuba'**/de OR **'djibouti'**/de OR **'dominican republic'**/de OR **'ecuador'**/de OR **'egypt'**/de OR **'el salvador'**/de OR **'eritrea'**/de OR **'equatorial guinea'**/de OR **'ethiopia'**/de OR **'fiji'**/de OR **'gabon'**/de OR **'gambia'**/de OR **'georgia (republic)'**/exp OR **'ghana'**/de OR **'grenada'**/de OR **'guatemala'**/de OR **'guinea'**/de OR **'guinea bissau'**/de OR **'guyana'**/de OR **'haiti'**/de OR **'honduras'**/de OR **'india'**/exp OR **'indonesia'**/exp OR **'iran'**/de OR **'iraq'**/exp OR **'jamaica'**/de OR **'jordan'**/de OR **'kazakhstan'**/de OR **'kenya'**/de OR **'north korea'**/de OR **'kosovo'**/de OR **'kyrgyzstan'**/de OR **'laos'**/de OR **'lebanon'**/de OR **'lesotho'**/de OR **'liberia'**/de OR **'libyan arab jamahiriya'**/de OR **'republic of north macedonia'**/de OR **'madagascar'**/de OR **'malaysia'**/exp OR **'malawi'**/de OR **'mali'**/de OR **'mauritania'**/de OR **'federated states of micronesia'**/de OR **'mauritius'**/de OR **'mexico'**/exp OR **'moldova'**/de OR **'mongolia'**/de OR **'montenegro (republic)'**/de OR **'morocco'**/de OR **'mozambique'**/de OR **'myanmar'**/de OR **'namibia'**/de OR **'nepal'**/de OR **'nicaragua'**/de OR **'niger'**/de OR **'nigeria'**/de OR **'pakistan'**/exp OR **'palau'**/de OR **'papua new guinea'**/de OR **'paraguay'**/de OR **'peru'**/de OR **'philippines'**/de OR **'romania'**/de OR **'russian federation'**/exp OR **'rwanda'**/de OR **'saint lucia'**/de OR **'saint vincent and the grenadines'**/de OR **'samoa'**/de OR **'sao tome and principe'**/de OR **'senegal'**/de OR **'serbia'**/exp OR **'sierra leone'**/de OR **'melanesia'**/de OR **'sri lanka'**/de OR **'somalia'**/exp OR **'south sudan'**/de OR **'sudan'**/de OR **'south africa'**/de OR **'suriname'**/de OR **'eswatini'**/de OR **'syrian arab republic'**/de OR **'tajikistan'**/de OR **'tanzania'**/de OR **'timor leste'**/de OR **'thailand'**/de OR **'togo'**/de OR **'tonga'**/de OR **'tunisia'**/de OR **'turkey (republic)'**/de OR **'turkmenistan'**/de OR **'uganda'**/de OR **'ukraine'**/exp OR **'ussr'**/exp OR **'uzbekistan'**/exp OR **'vanuatu'**/de OR **'venezuela'**/de OR **'viet nam'**/de OR **'yemen'**/de OR **'zambia'**/de OR **'zimbabwe'**/de

213,697

#1

'developing country'/exp OR **'developing country'** OR **'low income country'**/exp OR **'low income country'** OR **'middle income country'**/exp OR **'middle income country'** OR **'rural health'**/exp OR **'rural health'** OR **'rural population'**/exp OR **'rural population'**

3. Cochrane Library

Interface: Wiley<br><br>Date of Search: 31 March 2023<br><br>Number of hits: 8 (Reviews: 1, Trials: 7)	Field labels<br><br>• ti,ab,kw = title, abstract and author keywords<br>• NEAR/x = within x words, regardless of order<br>• * = truncation of word for alternate endings

ID Search Hits

#1 [mh ^"Medically Underserved Area"] OR [mh ^"Developing Countries"] OR [mh ^"Rural Health"] OR [mh ^"Rural Population"] 4285

#2 (Afghanistan:ti,ab,kw OR Albania*:ti,ab,kw OR Algeria*:ti,ab,kw OR "American Samoa":ti,ab,kw OR Angola*:ti,ab,kw OR Argentina:ti,ab,kw OR Armenia*:ti,ab,kw OR Azerbaijan:ti,ab,kw OR Bangladesh:ti,ab,kw OR Benin:ti,ab,kw OR Byelarus:ti,ab,kw OR Byelorussian:ti,ab,kw OR Belarus:ti,ab,kw OR Belorussian:ti,ab,kw OR Belorussia:ti,ab,kw OR Belize:ti,ab,kw OR Bhutan:ti,ab,kw OR Bolivia*:ti,ab,kw OR Bosnia*:ti,ab,kw OR Herzegovina:ti,ab,kw OR Hercegovina:ti,ab,kw OR Botswana:ti,ab,kw OR Brazil*:ti,ab,kw OR Brasil*:ti,ab,kw OR Bulgaria*:ti,ab,kw OR "Burkina Faso":ti,ab,kw OR "Burkina Fasso":ti,ab,kw OR Burundi:ti,ab,kw OR Urundi:ti,ab,kw OR Cambodia*:ti,ab,kw OR "Khmer Republic":ti,ab,kw OR Kampuchea:ti,ab,kw OR Cameroon:ti,ab,kw OR Cameroons:ti,ab,kw OR Cameron:ti,ab,kw OR Camerons:ti,ab,kw OR "Cape Verde":ti,ab,kw OR "Cabo Verde":ti,ab,kw OR "Central African Republic":ti,ab,kw OR Chad:ti,ab,kw OR China:ti,ab,kw OR Colombia*:ti,ab,kw OR Comoros:ti,ab,kw OR "Comoro Islands":ti,ab,kw OR Comores:ti,ab,kw OR Congo:ti,ab,kw OR "Costa Rica":ti,ab,kw OR "Cote d'Ivoire":ti,ab,kw OR "Ivory Coast":ti,ab,kw OR Cuba:ti,ab,kw OR Djibouti:ti,ab,kw OR Dominica:ti,ab,kw OR "Dominican Republic":ti,ab,kw OR "East Timor":ti,ab,kw OR "East Timur":ti,ab,kw OR "Timor Leste":ti,ab,kw OR Ecuador:ti,ab,kw OR Egypt*:ti,ab,kw OR "El Salvador":ti,ab,kw OR Eritrea*:ti,ab,kw OR "Equatorial Guinea":ti,ab,kw OR Eswatini*:ti,ab,kw OR Ethiopia*:ti,ab,kw OR Fiji:ti,ab,kw OR Gabon:ti,ab,kw OR Gambia*:ti,ab,kw OR Gaza:ti,ab,kw OR "Georgia Republic":ti,ab,kw OR "Georgian Republic":ti,ab,kw OR Ghana:ti,ab,kw OR Grenada:ti,ab,kw OR Guatemala*:ti,ab,kw OR Guinea:ti,ab,kw OR Guyana:ti,ab,kw OR Haiti:ti,ab,kw OR Honduras:ti,ab,kw OR India:ti,ab,kw OR Indonesia*:ti,ab,kw OR Iran:ti,ab,kw OR Iraq:ti,ab,kw OR Jamaica*:ti,ab,kw OR Jordan*:ti,ab,kw OR Kazakhstan:ti,ab,kw OR Kenya*:ti,ab,kw OR Kiribati:ti,ab,kw OR ("Democratic People's Republic of" NEXT Korea*):ti,ab,kw OR ("North" NEXT Korea*):ti,ab,kw OR Kosovo:ti,ab,kw OR "Kyrgyz Republic":ti,ab,kw OR "Lao PDR":ti,ab,kw OR Laos:ti,ab,kw OR Lebanon:ti,ab,kw OR Lesotho:ti,ab,kw OR Liberia*:ti,ab,kw OR Libya*:ti,ab,kw OR Macedonia*:ti,ab,kw OR Madagascar:ti,ab,kw OR Malaysia*:ti,ab,kw OR Malaya*:ti,ab,kw OR Malay:ti,ab,kw OR Malawi:ti,ab,kw OR Mali:ti,ab,kw OR Maldives:ti,ab,kw OR "Marshall Islands":ti,ab,kw OR Mauritania*:ti,ab,kw OR Mauritius:ti,ab,kw OR Mexico:ti,ab,kw OR Mehico:ti,ab,kw OR Micronesia*:ti,ab,kw OR "Middle East":ti,ab,kw OR Moldova:ti,ab,kw OR Moldovia*:ti,ab,kw OR Moldovian*:ti,ab,kw OR Mongolia:ti,ab,kw OR Montenegro:ti,ab,kw OR Morocco:ti,ab,kw OR Mozambique:ti,ab,kw OR Mocambique:ti,ab,kw OR Myanmar:ti,ab,kw OR Namibia*:ti,ab,kw OR Nauru:ti,ab,kw OR Nepal:ti,ab,kw OR Nicaragua:ti,ab,kw OR Niger:ti,ab,kw OR Nigeria*:ti,ab,kw OR Pakistan*:ti,ab,kw OR Palau:ti,ab,kw OR Palestine*:ti,ab,kw OR Paraguay:ti,ab,kw OR Peru*:ti,ab,kw OR Philippines:ti,ab,kw OR Philipines:ti,ab,kw OR Phillipines:ti,ab,kw OR Phillippines:ti,ab,kw OR Romania*:ti,ab,kw OR Rumania*:ti,ab,kw OR Roumania*:ti,ab,kw OR Russia:ti,ab,kw OR Russian:ti,ab,kw OR Rwanda:ti,ab,kw OR Ruanda:ti,ab,kw OR "Saint Lucia":ti,ab,kw OR "St Lucia":ti,ab,kw OR "Saint Vincent":ti,ab,kw OR "St Vincent":ti,ab,kw OR Grenadines:ti,ab,kw OR Samoa:ti,ab,kw OR "Samoan Islands":ti,ab,kw OR "Sao Tome":ti,ab,kw OR Senegal:ti,ab,kw OR Serbia*:ti,ab,kw OR "Sierra Leone":ti,ab,kw OR "Spanish Guinea":ti,ab,kw OR "Sri Lanka":ti,ab,kw OR

Karolinska Institutet

Ceylon:ti,ab,kw OR Solomon:ti,ab,kw OR USSR:ti,ab,kw OR "Soviet Union":ti,ab,kw OR "Union of Soviet Socialist Republics":ti,ab,kw OR Somalia*:ti,ab,kw OR ("South" NEXT Africa*):ti,ab,kw OR Sudan:ti,ab,kw OR Suriname:ti,ab,kw OR Surinam:ti,ab,kw OR Swaziland:ti,ab,kw OR Syria:ti,ab,kw OR "Syrian Arab Republic":ti,ab,kw OR Tajikistan:ti,ab,kw OR Tadzhikistan:ti,ab,kw OR Tadjikistan:ti,ab,kw OR Tadzhik:ti,ab,kw OR Tanzania:ti,ab,kw OR Thailand:ti,ab,kw OR Togo:ti,ab,kw OR "Togolese Republic":ti,ab,kw OR Tonga:ti,ab,kw OR Tunisia*:ti,ab,kw OR Turkey:ti,ab,kw OR Turkiye:ti,ab,kw OR Turkmenistan:ti,ab,kw OR Tuvalu:ti,ab,kw OR Uganda:ti,ab,kw OR Ukrain*:ti,ab,kw OR Uzbekistan:ti,ab,kw OR Uzbek:ti,ab,kw OR Vanuatu:ti,ab,kw OR Venezuela:ti,ab,kw OR Vietnam:ti,ab,kw OR "Viet Nam":ti,ab,kw OR "West Bank":ti,ab,kw OR Yemen:ti,ab,kw OR Zambia:ti,ab,kw OR Zimbabwe:ti,ab,kw) 101979

#3 ((developing:ti,ab OR emerging:ti,ab OR (less* NEXT "developed"):ti,ab OR "under developed":ti,ab OR underdeveloped:ti,ab OR "middle income":ti,ab OR (low* NEXT "income"):ti,ab OR third-world:ti,ab OR underserved:ti,ab OR "under served":ti,ab OR deprived:ti,ab OR poor*:ti,ab) NEAR/3 (countr*:ti,ab OR nation?:ti,ab OR population?:ti,ab OR world:ti,ab OR economy:ti,ab OR economies:ti,ab)) 9144

#4 (low*:ti,ab NEAR/2 (countr*:ti,ab OR gdp:ti,ab OR gnp:ti,ab OR "gross domestic":ti,ab OR "gross national":ti,ab)) 1401

#5 ((low*:ti,ab,kw OR middle*:ti,ab,kw) NEAR/5 (countr*:ti,ab,kw OR nation*:ti,ab,kw)) 4939

#6 ((rural:ti,ab OR remote:ti,ab OR nonmetropolitan:ti,ab OR non-metropolitan:ti,ab OR underserved:ti,ab OR "under served":ti,ab OR deprived:ti,ab OR shortage:ti,ab) NEXT (communit?:ti,ab OR count?:ti,ab OR area?:ti,ab OR region?:ti,ab OR province?:ti,ab OR district?:ti,ab)) 3392

#7 ("Global South":ti,ab,kw OR LIC:ti,ab,kw OR LMIC*:ti,ab,kw OR LMICs:ti,ab,kw OR MIC:ti,ab,kw OR South-South:ti,ab,kw OR ("rural" NEXT health*):ti,ab,kw OR ("rural" NEXT population*):ti,ab,kw) 6792

#8 #1 #2 OR #3 OR #4 OR #5 OR #6 OR #7 19039

#9 [mh ^"Hemorrhagic Fever, Ebola"] 132

#10 [mh ^Ebolavirus] 54

#11 [mh ^"Ebola vaccines"] 59

#12 [mh ^"Zika virus infection"] 53

#13 [mh ^"Zika virus"] 33

#14 [mh ^"Influenza A Virus, H1N1 Subtype"] 434

#15 (ebola:ti,ab,kw OR zika:ti,ab,kw OR zikv:ti,ab,kw OR h1n1*:ti,ab,kw) 1912

#16 (swine:ti,ab,kw AND (flu:ti,ab,kw OR influenza:ti,ab,kw)) 102

#17 [mh ^COVID-19] 4090

#18 [mh "COVID-19 Testing"] 112

#19 [mh "COVID-19 Vaccines"] 390

#20 (nCoV*:ti,ab,kw OR 2019nCoV:ti,ab,kw OR 19nCoV:ti,ab,kw OR COVID19*:ti,ab,kw OR COVID:ti,ab,kw OR SARS-COV-2:ti,ab,kw OR SARSCOV-2:ti,ab,kw OR SARS-COV2:ti,ab,kw OR SARSCOV2:ti,ab,kw OR "SARS coronavirus 2":ti,ab,kw OR "Severe Acute Respiratory Syndrome Coronavirus 2":ti,ab,kw OR "Severe Acute Respiratory Syndrome Corona Virus 2":ti,ab,kw) 15563

#21 #9 OR #10 OR #11 OR #12 OR #13 OR #14 OR #15 OR #16 OR #17 OR #18 OR #19 OR #20 17397

#22 [mh "HIV Infections"] 15863

#23 [mh HIV] 3733

#24 [mh Tuberculosis] 3263

#25 [mh Malaria] 4021

#26 [mh "Antitubercular Agents"] 2211

#27 [mh ^"Tuberculin Test"] 282

#28 [mh ^Antimalarials] 2015

#29 [mh "HIV testing"] 205

#30 [mh "Anti-HIV agents"] 4518

#31 (malaria*:ti,ab,kw OR antimalaria*:ti,ab,kw OR plasmodium:ti,ab,kw OR HIV:ti,ab,kw OR AIDS:ti,ab,kw OR tubercul*:ti,ab,kw OR antitubercul*:ti,ab,kw OR (immun* NEXT deficienc*):ti,ab,kw OR immunodeficienc*:ti,ab,kw) 52937

#32 #22 OR #23 OR #24 OR #25 OR #26 OR #27 OR #28 OR #29 OR #30 OR #31 52948

#33 #8 AND #21 AND #32 22

#34 (clinicaltrials or trialsearch):so 454244

#35 #33 NOT #34 8

4. Web of Science (Web of Science Core Colletion, CABI: CAB Abstracts & Global Health, Scielo)

Interface: Clarivate Analytics<br><br>Editions: WOS.SCI, WOS.AHCI, WOS.ESCI, WOS.SSCI, CABI.HEALTH, CABI.ABSTRACTS, SCIELO.SCIELO<br><br>Date of Search: 31 March 2023<br><br>Number of hits: 987	Field labels<br><br>• TS/Topic = title, abstract, author keywords and Keywords Plus<br>• NEAR/x = within x words, regardless of order<br>• * = truncation of word for alternate endings<br><br>Note: the *Exact search*-function was used for all the searches

#	Search Query	Results
1	TS=(Afghanistan OR Albania* OR Algeria* OR "American Samoa" OR Angola* OR Argentina OR Armenia* OR Azerbaijan OR Bangladesh OR Benin OR Byelarus OR Byelorussian OR Belarus OR Belorussian OR Belorussia OR Belize OR Bhutan OR Bolivia* OR Bosnia* OR Herzegovina OR Hercegovina OR Botswana OR Brazil* OR Brasil* OR Bulgaria* OR "Burkina Faso" OR "Burkina Fasso" OR Burundi OR Urundi OR Cambodia* OR "Khmer Republic" OR Kampuchea OR Cameroon OR Cameroons OR Cameron OR Camerons OR "Cape Verde" OR "Cabo Verde" OR "Central African Republic" OR Chad OR China OR Colombia* OR Comoros OR "Comoro Islands" OR Comores OR Congo OR "Costa Rica" OR "Cote d'Ivoire" OR "Ivory Coast" OR Cuba OR Djibouti OR Dominica OR "Dominican Republic" OR "East Timor" OR "East Timur" OR "Timor Leste" OR Ecuador OR Egypt* OR "El Salvador" OR Eritrea* OR "Equatorial Guinea" OR Eswatini* OR Ethiopia* OR Fiji OR Gabon OR Gambia* OR Gaza OR "Georgia Republic" OR "Georgian Republic" OR Ghana OR Grenada OR Guatemala* OR Guinea OR Guyana OR Haiti OR Honduras OR India OR Indonesia* OR Iran OR Iraq OR Jamaica* OR Jordan* OR Kazakhstan OR Kenya* OR Kiribati OR "Democratic People's Republic of Korea*" OR "North Korea*" OR Kosovo OR "Kyrgyz Republic" OR "Lao PDR" OR Laos OR Lebanon OR Lesotho OR Liberia* OR Libya* OR Macedonia* OR Madagascar OR Malaysia* OR Malaya* OR Malay OR Malawi OR Mali OR Maldives OR "Marshall Islands" OR Mauritania* OR Mauritius OR Mexico OR Mehico OR Micronesia* OR "Middle East" OR Moldova OR Moldovia* OR Moldovian* OR Mongolia OR Montenegro OR Morocco OR Mozambique OR Mocambique OR Myanmar OR Namibia* OR Nauru OR Nepal OR Nicaragua OR Niger OR Nigeria* OR Pakistan* OR Palau OR Palestine* OR Paraguay OR Peru* OR Philippines OR Philipines OR Phillipines OR Phillippines OR Romania* OR Rumania* OR Roumania* OR Russia OR Russian OR Rwanda OR Ruanda OR "Saint Lucia" OR "St Lucia" OR "Saint Vincent" OR "St Vincent" OR Grenadines OR Samoa OR "Samoan Islands" OR "Sao Tome" OR Senegal OR Serbia* OR "Sierra Leone" OR "Spanish Guinea" OR "Sri Lanka" OR Ceylon OR Solomon OR USSR OR "Soviet Union" OR "Union of Soviet Socialist Republics" OR Somalia* OR "South Africa*" OR Sudan OR Suriname OR Surinam OR Swaziland OR Syria OR "Syrian Arab Republic" OR Tajikistan OR Tadzhikistan OR Tadjikistan OR Tadzhik OR Tanzania OR Thailand OR Togo OR "Togolese Republic" OR Tonga OR Tunisia* OR Turkey OR Turkiye OR Turkmenistan OR Tuvalu OR Uganda OR Ukrain* OR	7267079

	Uzbekistan OR Uzbek OR Vanuatu OR Venezuela OR Vietnam OR "Viet Nam" OR "West Bank" OR Yemen OR Zambia OR Zimbabwe)	
2	(TI=((developing OR emerging OR "less* developed" OR "under developed" OR underdeveloped OR "middle income" OR "low* income" OR third-world OR underserved OR "under served" OR deprived OR poor*) NEAR/3 (countr* OR nation$ OR population$ OR world OR economy OR economies)) OR AB=((developing OR emerging OR "less* developed" OR "under developed" OR underdeveloped OR "middle income" OR "low* income" OR third-world OR underserved OR "under served" OR deprived OR poor*) NEAR/3 (countr* OR nation$ OR population$ OR world OR economy OR economies)))	336661
3	TI=(low* NEAR/2 (countr* OR gdp OR gnp OR "gross domestic" OR "gross national")) OR AB=(low* NEAR/2 (countr* OR gdp OR gnp OR "gross domestic" OR "gross national"))	37742
4	TS=((low* OR middle*) NEAR/5 (countr* OR nation*))	347043
5	TI=((rural OR remote OR nonmetropolitan OR non-metropolitan OR underserved OR "under served" OR deprived OR shortage) NEAR/0 (communit? OR count? OR area$ OR region$ OR province$ OR district$)) OR AB=((rural OR remote OR nonmetropolitan OR non-metropolitan OR underserved OR "under served" OR deprived OR shortage) NEAR/0 (communit? OR count? OR area$ OR region$ OR province$ OR district$))	159846
6	TS=("Global South" OR LIC OR LMIC* OR LMICs OR MIC OR South-South OR "rural health*" OR "rural population*")	223363
7	#6 OR #5 OR #4 OR #3 OR #2 OR #1	7645553
8	TI=(ebola OR zika OR zikv OR h1n1*) OR AB=(ebola OR zika OR zikv OR h1n1*) OR AK=(ebola OR zika OR zikv OR h1n1*)	49615
9	TI=(swine AND (flu OR influenza)) OR AB=(swine AND (flu OR influenza)) OR AK=(swine AND (flu OR influenza))	7479
10	TI=(nCoV* OR 2019nCoV OR 19nCoV OR COVID19* OR COVID OR SARS-COV-2 OR SARSCOV-2 OR SARS-COV2 OR SARSCOV2 OR "SARS coronavirus 2" OR "Severe Acute Respiratory Syndrome Coronavirus 2" OR "Severe Acute Respiratory Syndrome Corona Virus 2") OR AB=(nCoV* OR 2019nCoV OR 19nCoV OR COVID19* OR COVID OR SARS-COV-2 OR SARSCOV-2 OR SARS-COV2 OR SARSCOV2 OR "SARS coronavirus 2" OR "Severe Acute Respiratory Syndrome Coronavirus 2" OR "Severe Acute Respiratory Syndrome Corona Virus 2") OR AK=(nCoV* OR 2019nCoV OR 19nCoV OR COVID19* OR COVID OR SARS-COV-2 OR SARSCOV-2 OR SARS-COV2 OR SARSCOV2 OR "SARS coronavirus 2" OR "Severe Acute Respiratory Syndrome Coronavirus 2" OR "Severe Acute Respiratory Syndrome Corona Virus 2")	428480
11	#8 OR #9 OR #10	479886
12	TI=(malaria* OR antimalaria* OR plasmodium OR HIV OR AIDS OR tubercul* OR antitubercul* OR "immun* deficienc*" OR immunodeficienc*) OR AB=(malaria* OR antimalaria* OR plasmodium OR HIV OR AIDS OR tubercul* OR antitubercul* OR "immun* deficienc*" OR immunodeficienc*) OR AK=(malaria* OR antimalaria* OR plasmodium OR HIV OR AIDS OR tubercul* OR antitubercul* OR "immun* deficienc*" OR immunodeficienc*)	1005173
13	#12 AND #11 AND #7	3643
14	#12 AND #11 AND #7 and MEDLINE® (Exclude – Database)	987

Karolinska Institutet

5. Global Index Medicus

Interface: https://www.globalindexmedicus.net/<br><br>Date of Search: 31 March 2023<br><br>Number of hits: 631	Field labels<br><br>• tw = title, abstract, subject
tw:((tw:(malaria* OR antimalaria* OR plasmodium OR hiv OR aids OR tubercul* OR antitubercul* OR "immune deficiency" OR "immune deficiencies" OR immunodeficienc*)) AND (tw:(ebola OR zika OR zikv OR h1n1 OR swine OR ncov* OR 2019ncov OR 19ncov OR covid19* OR covid OR sars-cov-2 OR sarscov-2 OR sars-cov2 OR sarscov2 OR "SARS coronavirus 2" OR "Severe Acute Respiratory Syndrome Coronavirus 2" OR "Severe Acute Respiratory Syndrome Corona Virus 2"))) 631	
