# Supplementary material for: Interventions to Maintain HIV/AIDS, Tuberculosis, and Malaria Service Delivery During Public Health Emergencies in Low- and Middle-Income Countries: Protocol for a Systematic Review
Source: JMIR Res Protoc. 2025 Jan 15;14:e64316. doi: 10.2196/64316 (PMC11780283; doi:10.2196/64316)
Supplement: Multimedia Appendix 2 [file resprot_v14i1e64316_app2.docx]

**Appendix 2: Data Abstraction Form_Part 1**

| **#** | **Publication year** | **First Author** | **Year of study** | **Journal or source** | **Country(ies)** | **Title of article** | **Study design** | **Target population** | **Outbreak** | **Disease category** | **Intervention s implemented** |
| --- | --- | --- | --- | --- | --- | --- | --- | --- | --- | --- | --- |
|  |  |  |  |  |  |  |  |  |  |  |  |
|  |  |  |  |  |  |  |  |  |  |  |  |
|  |  |  |  |  |  |  |  |  |  |  |  |
|  |  |  |  |  |  |  |  |  |  |  |  |
|  |  |  |  |  |  |  |  |  |  |  |  |
|  |  |  |  |  |  |  |  |  |  |  |  |

**Data Abstraction Form_Part 2**

| # | **Author (Year)** | **Intervention** | **Measure of Assessment** | **Indicator Type** | **Service cascade** | **Implementation Level** | **Implementer** | **Assessment result** |
| --- | --- | --- | --- | --- | --- | --- | --- | --- |
